# Weather and Environmental Hazards at Mass Gatherings

**DOI:** 10.1371/4fca9ee30afc4

**Published:** 2012-07-31

**Authors:** Lee Soomaroo, Virginia Murray

## Abstract

Introduction Reviews of mass gathering events have traditionally concentrated on crowd variables that affect the level and type of medical care needed. Weather and environmental hazards at mass gathering events have not been fully researched. This review examines these events and aims to provide future suggestions for event organisers, medical resource planners, and emergency services, including local hospital emergency departments. Methods A review was conducted using computerised data bases: MEDLINE, The Cochrane Library, HMIC and EMBASE, with Google used to widen the search beyond peer-reviewed publications, to identify grey literature. All peer-review literature articles found containing information pertaining to lessons identified from mass gathering disasters due to weather or environmental hazards leading to participant death, injury or illness were analysed and reviewed. Disasters occurring due to crowd variables were not included. These articles were read, analysed, abstracted and summarised. Results 20 articles from literature search were found detailing mass gathering disasters relating directly to weather or environmental hazards from 1988 – 2011, with only 17 cases found within peer-review literature. Two events grey literature from 2011 are due to undergo further inquiry while one article reviews an event originally occurring in 1922. Analysis of cases were categorised in to heat and cold-related events, lightning and storms and disease outbreak. Conclusions Mass gathering events have an enormous potential to place a severe strain on the local health care system, Prior health resource and environmental planning for heat & cold-related illness, lightning & storms, and disease outbreak can advance emergency preparedness and response to potential disasters. Citation: Soomaroo L, Murray V. Weather and Environmental Hazards at Mass Gatherings. PLoS Currents Disasters. 2012 Jul 31 Keywords: Mass Gatherings, Disasters, Sporting Events, Festivals, Concerts, Storm, Lightning, Cyclone, Hot-weather illness, Cold-weather illness, Disease, Public Health, Syndromic Surveillance Abbreviations: ALS – Advance Life support; BLS – Basic Life support; ED – Emergency Department; EMS – Emergency Medical Services; PPR – Patient Presentation Rate

## Introduction

The definition of a mass gathering has previously been highlighted as an event at specific location of at least 1000 persons or more^1^ . This can also provide a strain to the planning and response resources of the local community, state or nation hosting the event (WHO, 2008). Mass gathering events, including scheduled events at sports facilities, air shows, rock concerts, outdoor celebrations, and visits by dignitaries, vary in their complexity and demand not only for medical service provision but also for emergency planning for all types of potential weather and environmental health hazards.

The United Nations International Strategy for Disaster Reduction (UNISDR) defines a hazard as “a dangerous phenomenon, substance, human activity or condition that may cause loss of life, injury or other health impacts, property damage, loss of livelihoods and services, social and economic disruption, or environmental damage”. If the hazardous event exceeds the ability of the affected community or society to cope using its own resources then it is defined as a disaster. In the context of this review of mass gathering incidents a weather or environmental hazard is to be considered as a natural event occurring suddenly causing great loss of life or damage to surroundings.

Historically, peer-review literature has concentrated on crowd variables that affect the level and types of medical need at a mass gathering event^2^ , studying patient presentation rates, and providing possible prediction tools for first-aid providers and local hospitals^3^ . However there is a lack of available evidence analysing weather or environmental hazards with future suggestions for event organisers, medical resource planners, and emergency services, including local hospital emergency departments.

This review aims to analyse previous cases of weather and environmental hazard disasters at mass gathering events, documenting the lessons identified to provide considerations when planning for future events. It is hoped, that with careful assessment of the mass gathering event as a whole it may be possible to plan ahead for potential number of attendees, consider health and safety aspects of planning for a mass gathering, plan for potential disasters and provide a more effective allocation of health and medical resources

## Methods

This literature review concentrates on extreme events and environmental health hazards at mass gathering events, focusing predominantly on case reports and literature reviews citing particular lessons identified from previous disasters.

A literature search was carried out using Medline, Cochrane, HMIC and Embase. The search terms are listed in Table 1. All peer-review literature articles found containing information pertaining to lessons identified from mass gathering crowd disasters were analysed and reviewed.

**Table 1. Key Literature Search words used in combination with Mass Gathering Events:** 

Weather Hazards	Geological Hazards	Disease
Drought	Avalanche	Water-bourne
Heat wave	Earthquake	Air-borne
Blizzard	Volcanic Eruption	Vector-bourne
Hailstorm	Tsunami	Food-bourne
Cyclonic Storms		
Ice strom		
Tornado		

A free-text search was also conducted using Google to link mass gathering events to disaster weather and environmental incidents in order to widen the search beyond peer-reviewed publications including grey literature (media reports, unpublished reports and commissioned inquiries).

Articles describing disasters occurring due to crowd variables such as analysis of cases categorised in to crowd control, event access, fire safety, medical preparedness and emergency response were excluded. Cases of events over a continual 12-month period were also excluded. Citations within articles were further searched to identify additional references that would inform this review.

It was decided not to include natural disasters having a major impact on local population such as the recent Earthquake and Tsunami of Japan and both earthquakes of New Zealand in 2011 as it was felt that these were mass casualty incidents, and not extreme events specific to mass gatherings.

Key words: Mass Gatherings, Disasters, Sporting Events, Festivals, Concerts, Storm, Lightning, Cyclone, Hot-weather illness, Cold-weather illness, Disease, Public Health, Syndromic Surveillance

## Results

In total, 20 reported mass gathering incidents from 1988 – 2011, were identified through the extensive literature search of weather and environmental hazards (Table 2), further highlighting the limited availability of mass gathering disaster reviews. While some reports provided valuable information regarding mass gathering disaster occurrence, most cases were descriptive rather than analytical, consequently very few literature reports were found to inform subjects of lessons identified from weather or environmental hazard disasters at mass gathering events. 17 of these incidents had been only reported in journal publications. Only three additional reported events were identified from grey literature searching, one reporting a building collapse from a snowstorm in 1922 and the other two related to mass gathering events in 2011.

**Table 2. Extreme Events & Environmental Health Risks at Mass Gatherings:** 

Date	Event-place	Environment	Health issues	Learning points identified	References
Jan 1922	Theatre Washinton USA	Snowstorm collapses theatre roof	98 deaths 133 injuries	• Structural integrity of the building • No early systems of MCI response	Zeman (2011) *Grey literature report
Feb 1988	Winter Olympics, Calgary, Canada	Cold weather conditions	PPR of 15.2 per 10,000	• 1% cold-related injury presentations • Mainly viral-related illness 24%	Thompson et al. (1991) *Review
Aug 1988	Music Festival, Michigan, USA	Shigellosis outbreak	3,175 cases of gastroenteritis	• Poor sanitation - no soap available • Limited access to running water • Very poor personal hygiene	Lee et al. (1990) *Case report
June 1991	Agriculture Show Adelaide, Australia	Warm weather conditions	Heat-related illness	Study findings Temperatures above 27°C are associated with a higher incidence of heat-related illness	Flabouris et al (1996) *Retrospective study
Aug 1996	Olympic Games, Atlanta, USA	Warm weather conditions	Heat-related illness	Public Health awareness • Increase public awareness • Encourage crowd to hydrate regularly • Identification of heat-related illness	Brennan et al. (1997) *Review
Aug 1996	Olympic Games, Atlanta, USA	Air pollution reduction program	Reduction in asthma-related ED presentation	Review findings Transport restrictions reduce the air pollution which contributes to reduction in respiratory related attendance	Friedman et al. (2001) *Review
June 1997	Music Festival, Glastonbury, UK	E. coli O157 from nearby live animals	7 presentations to local hospital	Recommendations • Hygiene information given to attendees • Cattle excluded from site 2 weeks prior • Chain harrowing of ground	Crampin et al. (1999) *Case report
July 1997	Football Match, Galicia, Spain	Hepatitis A from a public water fountain	23 cases	• Improve hygiene of fountain • Consider food and safety guidelines • Local health education	Abraria et al. (2000) *Case report
June 1998	Triathlon, Springfield, USA	Leptospirosis found in late water	23 cases	Study recommendation Empiric therapy with doxycycline, or a penicillin-based antibiotic should be considered for any suspicion of Leptospirosis.	Morgan et al. (2002) *Case report
Nov 1999	International fair, Kapellen, Belgium	Legionella from whirlpool aerosol spa	41 cases	• Water not changed during time of fair • Poor ventilation of surrounding area • Suggested cleaning with chlorine	De Schreiver et al. (2000) *Case report
June 1998	College Football, Washington, USA	Lightning strikes woman on cellphone	1 critically injured	Lightning safety recommendations • 30-30 Rule: (Table 4) • Weather early warning systems used • Evacuation protocol in place • Provision of shelters • Lightning safety tips given on flyers	Milzman et al. (1998) *Case report
Aug 2000	College Football, Virginia, USA	Lightning 1 km away from game	Mass evacuation No injuries	Lightning safety recommendations • 30-30 Rule: (Table 4) • Weather early warning systems used • Evacuation protocol in place • Provision of shelters • Lightning safety tips given on flyers	Hart (2000) *Grey literature report
Mar 2000	The Hajj, Mecca, Saudi Arabia	Outbreak of Meningococcal Disease W135	90 cases reported over 9 countries with 14 fatalities	• Public Health surveillance • Antibiotics offered to close contacts • Requirement to have vaccine prior to travel • No transmission rates following intervention	Aguiera et al. (2000) * Case report
Sep 2001	Swimming Gala, Sun-Moon Lake, Taiwan	Cold water conditions for swimmers	5 episodes of hypothermia	• Provision of upstream medical station • Poor communication links • Future: Provision of command centre	Chang (2001) *Review
2002	Agriculture Show, Adelaide, Australia	Heat-related illness	Review of 7000 patients over 7 years	Study Findings Positive correlation with number and type of casualty attendance with increased temperature and relative humidity	Zeitz et al. (2002)* Retrospective review
Feb 2002	Winter Olympics, Salt-Lake City, USA	Cold weather conditions	PPR of 26 per 10,000	• 3 cases of frostbite, no hypothermia • Mainly respiratory-related illness • Majority discharged home from site	Grissom et al. (2005) *Review
Jul 2005	College Football, USA	Warm weather conditions	Heat-related illness	Study Findings A 1 degree increase in temperature from 20°C to 21°C showed an 11% increase in patient attendance requiring medical attention	Kman et al. (2007) *Retrospective study
Nov 2005	College Football, Iowa, USA	Cyclone 15 km from game led to crowd diversion	Mass evacuation No injuries	Public Health Preparedness • Early Warning System • Shelters provided • Flyers given and announcements made • Game able to start following all-clear	Gallus (2006) *Case report
Aug 2008	Olympic Games, Beijing, China	Air pollution reduction programme	54% Reduction respiratory OPA presentation	Review Findings Transport restrictions reduce the air pollution which contributes to reduction in respiratory related attendance	Li et al. (2010) *Review
Mar 2009	Series of college events, USA	Heat-related illness and population	* Patient prediction model	• Suggestions for healthcare provision at event • Fails to prioritise environmental variables • Difficulty complex mass gathering prediction	Hartman et al. (2009) *Retrospective study
Aug 2011	State Fair, Indiana, USA	70mph winds tearing down stage	5 Deaths 45 Injured	*Commissioned Inquest • Storm occurred within 30 mins • Unclear if shelters were provided • Unknown structural integrity of stage	Guyett (2011) *Grey literature report
Aug 2011	Music Festival, Belgium	Hailstorm led to stage collapse	5 Deaths 65 Injured	*Commissioned Inquest • Storm occurred within 30 mins • Unclear if shelters were provided • Unknown structural integrity of stage	Blenkinsop (2011) *Grey literature report

20 reported mass gathering incidents have been read, analysed, abstracted, referenced and complied in chronological order and referenced (Table 2). The main learning points have been identified and further categorised into the following event triggers of heat (5), cold (4), lightning and storms (5), and disease outbreaks (6).

## Discussion

**Heat and cold-related events.** Heat-related illness and dehydration are amongst the commonest causes for patient presentations during mass gathering events, with a total of 5 identified.

A retrospective review of medical records from 47 college football games at two outdoor stadiums over a 5 year period found that a 1 degree increase in temperature from 20^O^C to 21^O^C showed an 11% increase in patient numbers requiring medical attention^4^. While a 1996 review of a 9-day Adelaide agricultural show found that high tempreatures greater than 27^O^C resulted in an increased patient presentation rate^5^ . This Same show was reviewed over a period of seven years concurred that patient presentation rates to on-site medical facilities increased in relation to relative tempreature and humidity^6^ . Emergency planners at the 1996 Atlantic Olympic Games were quick to increase the public awareness of to the dangers of dehydraton by encourgaing spectators to drink plenty of fluids, seek shade and to recognise the symptoms of dehydration resulted in a reduction of heat-related illness to medical centres, further highlighting to emergency planners that heat has a direct bearing on health provision at mass gatherings^7^ .

A patient presentation model was devised in 2009 which generates a scoring system based on the variables of weather, number of participants, presence of ethanol, crowd mood and age of the crowd in an effort to predict health resource utilisation at mass gathering events (Table 3). Despite its usefulness, it can be argued that the prediction tool fails to prioritise one variable over another and that it also hinders the ability to make predictions about specific or complicated mass gatherings^8^ .

**Table 3A. Prediction of patient presentation rates at Mass Gathering Events.** Adapted from Hartman et al: Am J Emerg Med 2009 Mar;27(3):337-43.

Weather (Heat Index)	Crowd attendance	Ethanol	Crowd age	Crowd intention	Point value
> 32.2º C	> 15,000	Significant	Older	Animated	2
< 32.2º C Climate not controlled	1,000- 15,000	Limited	Mixed	Intermediate	1
Climate controlled	< 1,000	None	Supervised younger	Calm	0

**Table 3B. Prediction of patient presentation rates at Mass Gathering Events.** Adapted from Hartman et al: Am J Emerg Med 2009 Mar;27(3):337-43.

Event classification	Score & recommendations
Major	Total Score > 5 or Scores of 2 in 2 different categories (Multiple ALS personnel, Specialised equipment, Physicians)
Intermediate	Total Score > 3 but < 5 or Score of 2 in any 1 category (2 transport units with 1-3 ALS & 1-6 BLS providers)
Minor	Total Score < 3 (Single transport vehicle with 1 ALS & 1 BLS provider)

Four cold related adverse health impacts at mass gathering events were identified.

Cold-related events have found low patient presentations rates to on-site medical centres, with an incidence of 15.2 per 10,000 people during the Winter Olympics in Calgary 1988^9^ and, 26 per 10,000 people at the Winter Olympics in Salt-Lake City 2002^10^ . Both studies found the main request for medical assistance was linked respiratory symptoms. A study of the Sun-Moon Lake Swimming Carnival in 2010 yielded five cases of hypothermia over a long-distance swim of 3.3km. The on-site medical care was found to be adequate, but extra medical facilities along the swimming course were suggested, with the provision of a medical command centre on site. It was also noted that across the entire event site communication between medical centres and organisers was poor^11^ .

A 1998 article of a 1922 snowstorm was found from newspaper archives. Although it is not possible to confirm that the numbers fit with the mass gathering definition given above it is considered of interest to include this historic report in this case series. Following heavy snowfall overnight on 29 January 1922, the Knickerbocker Theatre in Washington DC suffered extensive storm damage. The heavy snow on top of the theatre became too much for the roof to handle, it began to give way. The roof collapsed on top of audience members watching a silent comedy resulting in 98 deaths and 133 injuries. At the time there was no on-site health care provision, emergency triage, or mass casualty incident planning. Improvements in building safety as well as international medical triage and mass casualty care have further served to limit future incidences like this^12^ .

Through previous literature, it appears that heat and cold-related illness provide the highest number of patient presentation to hospitals following mass gathering events. This can be linked to seasonal analysis to enable event and emergency planners to provide adequate medical care ensuring:

- Provision of on-site medical care to cope with potential crowd size
- On-site health promotion to avoid heat or cold related illness
- Potential use of scoring systems to predict patient presentation
- Emergency planning to incorporate mass casualty incidents

**Lightning & Storms.** A total of five mass gathering related events were identified in the search strategy.

Large outdoor stadiums face a significant and growing vulnerability. Two case reports were found that relate to mass gatherings and the effect of lightning. One woman in 1998 was struck by lightning while talking on her cellular telephone while at stadium concert inWashington DC, USA. She went on to survive despite several minutes of CPR provided by two by-standers who happened to be a trauma paramedic and a surgical resisdent^13^ . While a newspaper column in 2000 reported how a lightning strike 1km away from a College football stadium prompted organisers to quickly cancel a game. Stadium management and game officials at the time were unable to communicate to the crowd due to an inadequate loudspeaker system causing fans to crowd the exit tunnels in panic^14^ .

Despite recent advances in meteorological technology and the advancement of early warning systems, A 30-30 rule to lightning safety involving simple observation of local weather (Table 4) has the potential to be utilised at future mass gathering events^15^ .

**Table 4. The 30-30 Rule for Lightning Safety at Outdoor Events:** Adapted from - Holle et al: Bull Amer Meteor Soc.;1999; 80:2035–2042

1. A flash-to-bang (lightning to thuder) count of 30 seconds indicates lightning 10 km away. activity should be suspended and crowd moved to designated shelters.
2. Wait 30 minutes after the last lightning or thunder berfore restarting the event.

The formation of lightning and severe weather safety guidelines and an potential provision of a stadium action plan as perviously been suggested including^16^ :

- Assessment of stadium protection and hazard vulnerability
- Use of weather early warning systems
- An evacuation protocol to designated shelters
- Crowd safety and evacuation tips posted on flyers and programmes

These ideas may have already been used in combination to avert a further disaster on 21 November 2005. At the last home football match of Iowa State University, Officials had known for several days in advance about a severe weather threat. Prior to kick-off a Tornado was located roughly 15 miles away from the event. Flyers and large TV screens were used to inform fans of the impeding weather threat. An evacuation of the stadium was ordered and fans were ushered to safety shelters. The cyclone passed within 3 miles of the event. The game started 40 minutes after the all-clear was given^17^ .

Unfortunately, despite provision of early warning systems, devastating storms can strike at a moments notice. On August 14 2011 a stage at a fair in Indianna USA collapsed killing 5 and injuring 45 after 70mph winds with rain and hail were reported to have torn down the structure trapping people below, only minutes after a safety announcement was given^18^ . Failure to share National Weather Service predictions about an approaching storm, reluctance of the band to delay its performance, and a stage structure too weak to resist the winds' lateral force were cited as factors found by the Indianna State Commision.

A few days later on August 19 2011, a strong hailstorm contributed to the death of 5 people and a further 70 injured as a stage collapsed at the Pukkelpop festival in Belgium. The storm was reported earlier in the evening as sweeping across Belgium, rapidly turning the sky dark over a period of 30 minutes^19^ , which was later discovered to be a Supercell thunderstorm, characterised by a sudden persistently-rotating updraft of air (mesocyclone) capable of producing severe weather combination including high winds, large hail, and strong tornadoes.

Both cases highlight the sudden onset of storm behaviour. The average lead time for warning (the amount of time between the issuance of a warning and the touchdown of severe weather) of ‘supercell’ thunderstorms according to the National Severe Storms Laboratory is currently 11 minutes^20^ , making severe weather difficult to predict and a large scale evacuation of a mass gathering in a short space of time a major challenge to planning a mass gathering event.

**Disease Outbreak.** A total of six adverse health effects from mass gathering events were reported in the peer review literature.

An early case of a Shigella outbreak from uncooked tofu was found at a major outdoor music festival in 1988. Poor sanitation available on site with limited access to running water was thought to be the cause^21^ . While an outbreak of Legionella during a fair in Belgium noted the source to be a aerosolised whirlpool spa. The study found that the water during the fair was not changed, with poor ventilation of the surrounding area. It was suggested that chlorine should be used for further fairs to clean the tanks. The need to consider provision of clean water facilities and water hygiene is important when planning for a mass gathering event.

23 cases of Hepatitis A were reported in a province of Spain during a soccer championship. Following epidemiologic and environmental investigation, the source of the outbreak was identified as water consumed from a village water fountain^23^ .

Lake water was identified as the source of 23 cases of Leptospirosis during a triathalon in 1998. Following this it was advised that empirical antibiotic therapy with doxycycline, or a penicillin-based antibiotic should be considered for any presentation of those suspected with Leptospirosis after exposure to outdoor freshwater sources^24^ .

Infection from live animals exists as a possibility whenever they are in close proximity to a mass gathering event and should also have planning consideration. At the Glastonbury festival UK in 1997, 7 cases of E. Coli O157 presented to hospital, which was attributed to contact with local livestock. Since the event, local organisers have given hygiene recommendations to attendees and cattle are excluded from the site for 2 weeks beforehand^25^ .

The Centre for Disease Control and Protection (CDC) in Atlanta advocates that ‘Public health surveillance should be implemented at mass gathering events to facilitate rapid detection of outbreaks and other health-related events and enable public health teams to respond with timely control measures’. An important example of syndromic surveillance was described following an outbreak of Meningococcal disease W135 Serogroup at the Hajj in 2000. Over 90 cases in 9 countries were found, with 14 deaths. In France and the UK, patients were contacted and treated with antibiotics, with further prophylactic antibiotics given to those in close contact with patients. From 2001, the health organisers of the Hajj declared that all pilgrims are required to have taken the quadrivalent meningococcal vaccine prior to travelling to the Saudi Arabian region^26^ .

## Conclusion

Each mass gathering event has its own separate risk of extreme weather behaviour and environmental health hazards. Literature on the adverse health effects arising from any mass gathering events is extremely limited with learning points very difficult to identify. However, from the detailed analysis of previous weather or environmental incidents in this review the following learning points for emergency and health resource planners can be applied:

- Use of weather surveillance and severe weather early warning systems
- Medical facilities stocked for a range of weather or environmental hazards
- Provision of adequate, shade, water facilities and first aid on site
- Medical care provided by a multi-professional team in relation to crowd size
- Food and water hygiene and safety regulations followed
- Local livestock evaluated for potential disease transmission
- Local public health inclusion in event planning to promote health awareness and provide a basis for post-event syndromic surveillance.

Further studies highlighting the impact of weather and environment hazards to health and their effect on patient presentation to medical centers at mass gathering events and local hospitals are warranted to advance not only the epidemiological knowledge base, but to also gain further lessons of emergency preparedness and response.
